# Secondary antibody therapy outperforms corticosteroids in an ameliorating lipopolysaccharide-induced rat model of premature ovarian failure

**DOI:** 10.17221/66/2025-VETMED

**Published:** 2026-02-27

**Authors:** Abdelkader A. Zaki, Saleh M. Albarrak

**Affiliations:** ^1^Department of Medical Biosciences, College of Veterinary Medicine, Qassim University, Buraydah, Saudi Arabia; ^2^Department of Physiology, College of Veterinary Medicine, Cairo University, Cairo, Egypt; ^3^Department of Pathology and Laboratory Diagnosis, College of Veterinary Medicine, Qassim University, Buraydah, Saudi Arabia

**Keywords:** antibodies, autoimmunity, ELISA, frankincense, glucocorticoids

## Abstract

Premature ovarian failure (POF) is a significant cause of infertility and is often linked to autoimmune aetiologies. Lipopolysaccharide (LPS)-induced inflammation is a well-established model of autoimmune POF in rodents. Immunomodulatory treatments involving corticosteroids, frankincense, and targeted secondary antibodies have been hypothesised to mitigate the autoimmune response, reduce anti-ovarian antibody (AOA) levels, and restore ovarian function in an LPS-induced POF rat model. A POF model was established in female albino rats via the intraperitoneal injection of LPS. The rats were then divided into groups that received no treatment (LPS control), dexamethasone (DEX-treated LPS-treated rats), methylprednisolone (MP-treated LPS-treated rats), frankincense (Frankincense-treated LPS-treated rats), or secondary anti-ovarian antibodies (secondary Ab-treated LPS-treated rats) for 3 to 4 weeks. The serum levels of AOA, 17β-oestradiol, follicle-stimulating hormone (FSH), and luteinising hormone (LH) were assayed via commercial enzyme-linked immunosorbent assay (ELISA) kits. Ovarian tissues were examined histopathologically to assess structural damage and recovery. LPS induction successfully created a POF phenotype, as evidenced by significantly elevated AOA levels (*P*** < **0.001), reduced 17β-oestradiol (*P*** < **0.001), elevated FSH/LH (*P*** < **0.001 and *P*** < **0.05, respectively), and severe histopathological damage, including follicular atresia. All the treatments restored 17β-oestradiol levels. Secondary antibody therapy was most effective, normalising all hormonal parameters, significantly reducing AOA levels, and demonstrating complete histological recovery with healthy follicles and corpora lutea. MP potently suppressed AOA but paradoxically elevated FSH, without improving ovarian histology. DEX and frankincense showed intermediate efficacy, improving some hormonal and serological markers but failing to achieve full histological restoration. These findings demonstrate that targeted immunotherapy using secondary antibodies is superior to broad immunosuppression or anti-inflammatory treatment for restoring ovarian function in patients with autoimmune POF. While corticosteroids effectively reduce AOA titres, they may not reverse ovarian damage and can disrupt the hormonal balance. This underscores the need for precise, biomarker-guided therapies over nonspecific immunosuppression in patients with autoimmune ovarian insufficiency.

Recently, interest in reproductive immune infertility has increased (Al Ramadan et al. 2021). Although the ovary is not an immunologically favoured organ, fertility suffers greatly when tolerogenic mechanisms for ovarian-specific antigens fail. The ovary and its vessels can be targets of an autoimmune attack under various circumstances (Saeed 2017). Abdominal or pelvic inflammatory processes may include inflammation of the ovaries (ovaritis) or the ovarian stroma, particularly the oocytes (oophoritis). Accurate assessment of the prevalence of the autoimmune form of infertility has been challenging owing to the lack of sensitive and specific diagnostic tools. Mammals with ovaries and early ovarian failure are known to be affected by autoimmune diseases (Shoukry et al. 2020).

Ovarian hypofunction, along with increased FSH and decreased E2 levels, is known as premature ovarian failure (POF), a heterogeneous illness (Flatt et al. 2023). The pathophysiology and aetiology of autoimmune-mediated POF are still poorly understood, according to Ishizuka (2021), even though the ovary was first recognised as an autoimmunity target. Because gonadotropin and steroid hormones regulate folliculogenesis in all animals and anomalies result in ovarian aberrant diseases such as POF, they are reliable predictors of POF.

Anti-ovarian antibodies (AOA) may aid in the identification of ovarian autoimmunity (Makled et al. 2015). AOA are a class of autoantibodies that target various ovarian antigens. The harvest of oocytes for use in assisted reproductive procedures, minimally invasive surgery, and chronic ovaritis are only a few of the causes of AOA development (Al-Naffakh and Risan 2020). The pathological significance of AOA to various ovarian cell components can decrease pregnancy and fertilisation rates, in addition to creating a poor response to gonadotropin induction, and could be responsible for implantation failure (Kirshenbaum and Orvieto 2019). AOA targets the ovaries, making them malfunctional, preventing them from generating the hormone oestrogen normally, or causing the release of eggs to be irregular. The AOA is an independent predictor of the start of ovarian failure, according to Thabbah et al. (2015). Since AOA are known to be related to poor outcomes in subfertility, screening for their existence before the start of treatment may be advised.

Immunosuppressive medications are typically used in the conventional treatment of autoimmune disorders. Experts continue to debate whether to treat autoantibodies such as AOA with corticosteroids, given conflicting studies. Williams et al. (2008) reported that ovarian cell activity is inhibited by both lipopolysaccharide (LPS) and tumour necrosis factor alpha. According to Magata (2020), cows with uterine inflammation may experience infertility as a result of the harmful effects of LPS on ovarian function. According to Lv et al. (2021), a mouse model of LPS-induced primary ovarian insufficiency was successfully established, and LPS stimulation led to granulosa cell death, fibrosis, and ovarian inflammation. In the present study, AOAs were produced via an albino rat model of POF caused by LPS.

The present study aimed to clarify the effects of three treatments on circulating AOA levels and ovarian activity in an LPS-induced POF rat model. POF rats were administered dexamethasone (DEX), methylprednisolone (MP), frankincense, or secondary antibodies. DEX and MP are immunosuppressive corticosteroids (Mehta et al. 2022). Oleogum resins, or frankincense, are extracted from thick Boswellia trees, which grow in desert areas (Al-Yasiry and Kiczorowska 2016). Efferth and Oesch (2022) used it as an anti-inflammatory medication. Secondary antibodies are therapeutic antibodies specific to ovarian antibodies; this is likely because the primary “foreign” element of the injected antibody molecule is the idiotype component (Großerichter-Wagener et al. 2020). This study highlights the roles of AOAs in infertility and explores potential therapeutic approaches for AOA suppression and the restoration of ovarian activity. DEX and MP were selected as representative corticosteroids to compare their immunosuppressive efficacy and potential differential effects on ovarian recovery. Frankincense is included as a natural anti-inflammatory agent with historical and emerging scientific support. Secondary antibodies were chosen to evaluate a targeted immunotherapeutic approach aimed specifically at neutralising pathogenic anti-ovarian antibodies.

## MATERIAL AND METHODS

### Ethical approval

All the study methods were performed by the Animal Care and Use Committee of the Experi-mental Animal Centre at Qassim University, No. 22-15-11. Every attempt was made to decrease pain.

### Preparation of rat ovarian antigens and primary and secondary antibodies

Ovarian tissues were collected from adult albino rats, homogenised, and centrifuged to obtain protein-rich supernatants, which were used as immunisation antigens. Primary antibodies were raised in New Zealand rabbits by subcutaneous immunisation with an ovarian protein emulsion, using the method of Chiu and Chamley (2004), against known supernatant proteins. Immunoglobulins were isolated via ammonium sulphate precipitation and dialysis. The secondary antibodies were produced by immunising additional rabbits with the primary antibodies following a similar protocol.

### Checkerboard titration

The optimal antigen dilution and effectiveness of the rabbit-made protein antisera were assessed via checkerboard titration. Serial dilutions of the tested antigen were used to coat the ELISA plate, and negative control serum was utilised to compare various dilutions of the tested antisera. The serum dilution with the lowest concentration of antigen/well that produced a clear signal was considered the optimal condition and was used in subsequent tests at the known dilutions of conjugate and substrate. When the efficacy of the rabbit antisera against the antigen (300 ng of sperm protein) was tested, the optimal dilution was 1 : 32. The conventional log-dose response curve of rat protein was used to calculate the volume of rat protein used.

For sheep anti-rabbit IgG (Product No. A-5279; Sigma Co., St. Louis, U.S.A.) and sheep anti-rat IgG (Product No. A-5287; Sigma Co., St. Louis, U.S.A.), the optimal dilutions were 1 : 1 000 and 1 : 800, respectively. After two ELISA plates (Nunc, Roskilde, Denmark) with serial dilutions of the tested ovarian protein diluted with carbonate bicarbonate buffer (0.159% sodium carbonate and 0.293% sodium bicarbonate, pH 9.6) were coated, negative control serum and various dilutions of the tested hyperimmune serum were added.

Serum dilution with the lowest ovarian protein concentration/well that produced a detectable signal will be used in future testing under known conjugate and substrate dilutions, as this was determined to be the best condition. On the basis of the effectiveness of the main antibody against 300 ng of ovarian protein, the optimal dilution was found to be 1 : 16. The potency of the rat ovarian protein antisera produced in the rabbit curve indicates the quantity of antigen needed. Following the application of ovarian protein sex point dilutions ranging from 250 ng to 15.62 ng (one dilution per row) to the plate, the antigen was utilised as the negative control.

The plate was loaded with 100 μl/well of Ab1 (1: 16 in PBS) after three rounds of washing with washing buffer (pH 7.2, 0.05% Tween-20), and it was shaken for 1 h at 37 °C. The plate was incubated for an additional hour at 37 °C in a shaking water bath after being filled with 100 μl/well of sheep anti-rabbit IgG conjugates (1 : 2 000 dilution in PBS, pH 7.2). Following incubation, the plate was rinsed three times before 100 μl of TMB as a substrate solution (3,3'5,5'-tetramethylbenzidine) (30 mg) in 75 ml Dw (Sigma Chemical Co.) was added to each well. After the plate was left at room temperature in the dark for 7 min, the reaction colour became visible.

To halt the reaction, 50 μl/well of the stop solution (2.5 M H_2_SO_4_) was added. A microplate ELISA reader (ELx800UV; BIO-TEK, INC., Winooski, VT, USA) was used to measure the enzyme-mediated reaction at 492 nm. The association between optical density and log antigen dosage was then displayed as a standard curve. With the exception of substituting ovarian proteins with five consecutive dilutions of Ab2 (5–0.5 μg) and then adding the negative control, the procedures were the same for the checkerboard titration of the secondary antibody as an antigen.

The optical density was then plotted against the log-dose of secondary antibody as the antigen to create a standard curve. The formula optical density (*y*) = 0.355 4 + 0.126 9 log-titre (*X*) demonstrated the potency of the generated rat ovarian protein antisera in rabbits, with an *R*-squared value of 96.3% and an *R*-Sq adj. of 96.4%. The optical density (*y*) = –0.233 4 + 0.237 5 log-conc(*X*) was the normal log-dose response curve for the rat protein antibody, with an *R*-squared value of 97.5% and an *R*-Sq adj. of 97.0%. The generated Ab2 has a strong optical density (*y*) = –1.111 + 1.321 log-conc(*X*), an *R*-squared value of 93.1%, and an *R*-Sq adj. of 91.4%.

### Experimental design

Daily vaginal smears were performed prior to administration, and the vaginal epithelial cells were analysed to evaluate changes in the oestrous cycle. The dropper is carefully placed into the rat’s vagina, and saline is pumped into the vagina and then removed again to collect vaginal epithelial cells. Dioestrus, proestrus, oestrus, and metestrus, the four regular phases of the oestrous cycle, were recognised and chosen. Eight rats were fed and allowed to drink normally without any change and served as the negative control (NC) group. A premature ovarian failure (POF) rat model was generated by intraperitoneal injection of lipopolysaccharide (LPS). Forty rats were split into five equal treatment groups and given intraperitoneal injections of 0.5 mg/kg LPS (Sigma Aldrich, St. Louis, MO, USA) once daily for 14 days to induce POF (Wang et al. 2020; Lv et al. 2021). The treatment groups were as follows: LPS-treated rats (positive control) were fed and allowed to drink normally without any medicinal intervention. DEX-treated LPS rats: For 3 consecutive weeks, this group received 2 weekly intramuscular injections of DEX sodium phosphate (2.25 mg/kg) from Egyptian INT. Pharmaceutical Industries CO. E.I.P.I.C.O. (Earp et al. 2008).

Anti-ovarian antibody-treated LPS-treated rats in the second group were injected intraperitoneally with 50 mg/kg body weight secondary anti-ovarian antibodies prepared via the same regimen as DEX. Methylprednisolone (MP)-treated LPS-treated rats: Metraprednisolone (NUPCO/MEDIS BP 206-8000 Nabeul – Tunisie Batch No. 21F 0207) was injected intramuscularly at a dose of 2.25 mg/kg via the same regimen as DEX (Cavus et al. 2014). In accordance with Al-Marhoon et al. (2023), frankincense male gum was extracted from Hojari, Salalah, and Sultanate Oman at a concentration of 200 mg/kg/day. It was then utilised as a raw material, powdered in a mortar and dissolved in distilled water at a concentration of 1 g/30 ml d.w. Each rat received 100 mg per day for 30 days. Using a stomach tube, the rats were given frankincense gum orally. Before beginning the procedures for the AOA examination (day zero), blood samples were obtained from the inner canthus of the rats. For the four weeks of the experiment, blood samples (3 ml/animal) were taken every two weeks to obtain serum.

### E2 FSH and LH concentrations

The concentrations of various plasma hormones were measured in triplicate using commercial enzyme-linked immunosorbent assay (ELISA) kits according to the manufacturers’ instructions. Specifically, oestradiol levels were determined using a kit with a sensitivity of 8.7 pg/ml and a detection range of 21.23–571.44 pg/ml (Estradiol ELISA kit, 17 oestradiol antigenic; Monocent, Inc., Canoga Park, CA, USA). Both follicle-stimulating hormone (FSH) and luteinising hormone (LH) were assessed via rat-specific ELISA kits (Elabscience, USA; Cat. No. E-EL-R0391 and EL-R0026, respectively). The FSH assay had a sensitivity of 0.1 ng/ml and a range of 0.6–40 ng/ml, with intra- and interassay CVs of 10% and 12%, respectively. The LH assay had a sensitivity of 1.88 ng/ml, a range of 3.13–200 ng/ml, and intra- and interassay CVs below 10%.

### Histopathological examination

Ovarian tissues were fixed in 10% neutral buffered formalin, embedded in paraffin, sectioned at 5 μm, and stained with haematoxylin and eosin (H&E) for light microscopic evaluation. Histopathological changes, including follicular atresia, degeneration, and corpus luteum integrity, were assessed.

### Measurement of AOA concentrations in rat serum

The concentration of immunoreactive anti-ovarian antibodies (AOAs) was quantified via an indirect enzyme-linked immunosorbent assay (ELISA), adapted from the methodology established by Esmailnejad et al. (2020). The procedure was conducted as follows: First, the solid phase was prepared by coating each well of a microplate with 100 μl of a solution containing 300 ng of rat ovarian protein in carbonate-bicarbonate buffer. The plate was then sealed with adhesive tape to prevent evaporation and shaken in a 37 °C water bath for two hours to ensure uniform coating. The plate was subsequently incubated overnight at 4 °C to facilitate complete protein adsorption to the well surface. After incubation, any unbound ovarian proteins were removed by washing the plate three times with an appropriate buffer. To prevent nonspecific antibody binding, the remaining vacant sites in the wells were blocked with 200 μl of blocking buffer (2% bovine serum albumin, pH 7.2). The plate was sealed and shaken again at 37 °C for two hours. A series of three washes was subsequently performed to clear the blocking buffer. Next, 100 μl of each serially diluted test serum sample was added to the wells, and the plate was incubated at 37 °C with shaking for one hour. After three additional washes to remove unbound antibodies, 100 μl of sheep anti-rat IgG conjugate was added to each well. This was followed by a further one-hour incubation at 37 °C with shaking. After a final set of three washes, 100 μl of tetramethylbenzidine (TMB) substrate buffer was added to each well to initiate the enzymatic reaction. The plate was then kept in the dark at room temperature for seven minutes to allow colour development. The reaction was stopped by adding 50 μl of a stop solution to each well. The intensity of the resulting colorimetric signal, proportional to the AOA concentration, was finally measured using a microplate ELISA reader at 492 nm.

In biological research, ELISA is a standard diagnostic tool used to detect specific antibodies and classify samples as positive or negative. For this study, six readings were recorded for each sample. The corresponding ELISA values for the rat samples across the different experimental groups are presented in Table 1. The process began by measuring the optical densities (ODs) of undiluted samples. These samples were then diluted across the wells of an ELISA plate. The calibrated concentration for the average undiluted sample was determined. A critical cut-off value was established for each assay using the following formula: mean OD of the negative control + 3 standard deviations (Kumar and Rao 1991). This cut-off value was then used to find a corresponding calibrated concentration from a standard dose–response curve. The antibody titre for a sample was defined as the mean dilution factor at which its OD value fell below the calculated cut-off endpoint. A sample was classified as positive if its dilution point was above the cut-off value and negative if it was below. Finally, an antibody index was calculated for each sample to quantify its reactivity relative to the controls: (OD of the sample – OD of the negative control)/(OD of the positive control – OD of the negative control) × 100, as described by Bauer et al. (2002).

**Table 1:** Estimated ELISA readings

Animal grouping	Sampling time	OD of intact samples	Concentration of intact samples (ng/100 μl)	OD of the cut-off endpoint	Concentration at cut-off endpoint (ng/100 μl)	Antibody titre at cut-off values (dilution at cut-off endpoint)	Antibody index (%)
Normal rats (negative control)	Wk2	0.136 ± 0.001	5.757 ± 0.044	0.036 ± 0.000 4	3.79 ± 0.323	6.50 ± 1.50	6.499 ± 0.109
	Wk4	0.050 ± 0.007	3.728 ± 0.142	0.05 ± 0.077	0.261 ± 0.071	13.05 ± 4.18	15.30 ± 5.00
							
LPS-rats (positive control)	Wk2	0.771 ± 0.236	59.3 ± 15.6***	0.073 ± 0.012	36.85 ± 8.18	53.3 ± 10.7***	94.17 ± 5.59***
	Wk4	0.431 ± 0.070	48.5 ± 46.50***	0.190 ± 0.050	19.273 ± 0.739	85.3 ± 21.30***	80.89 ± 4.35***
							
MP-treated LPS-rats	Wk2	0.446 ± 0.115	42.012 ± 5.90^a^	0.051 ± 0.007	17.348 ± 0.324	4.50 ± 1.26^c^	28.21 ± 8.05^c^
	Wk4	0.296 ± 0.070	33.40 ± 4.84^a^	0.054 ± 0.007	17.46 ± 0.092	6.50 ± 1.50^c^	17.76 ± 4.92^c^
							
Frankincense -treated LPS-rats	Wk2	1.000 ± 0.209	78.00 ± 14.00	0.057 ± 0.017	24.24 ± 7.41	44.0 ± 12.00	88.2 ± 14.7
	Wk4	0.780 ± 0.093	85.6 ± 21.90	0.037 ± 0.002	16.708 ± 0.091 0	74.00 ± 12.00	51.71 ± 6.60
							
DEX-treated LPS-rats	Wk2	0.534 ± 0.106	68.9 ± 13.8	0.040 7 ± 0.003 2	16.748 ± 0.255	5.50 ± 1.50^c^	65.63 ± 3.11^a^
	Wk4	1.091 ± 0.074	94.1 ± 14.30	0.027 ± 0.002	16.310 ± 0.031 9	54.00 ± 12.00	48.05 ± 4.53^a^
							
Secondary abs-treated LPS-rats	Wk2	0.307 ± 0.025	31.22 ± 4.95^a^	0.037 5 ± 0.000 8	16.738 ± 0.102	10.00 ± 2.00^c^	45.7 ± 5.97^a^
	Wk4	0.260 ± 0.085	18.6 ± 6.90^b^	0.038 0 ± 0.001 7	16.770 ± 0.072 2	14.00 ± 2.00^c^	50.30 ± 6.29^a^
							
*P-*values		<0.646	<0.002	<0.163	<0.333	<0.000	<0.002

^a–c^Different lowercase letters indicate significant differences compared with the positive control group: *P* < 0.05, *P* < 0.01, and *P* < 0.001, respectively. The data are presented as the means ± standard errors (SEs). Asterisks denote significant differences compared with the negative control group: *P < 0.05, **P < 0.01, ***P < 0.001

DEX = dexamethasone; LPS = lipopolysaccharide; MP = methylprednisolone; OD = optical density

This study measured anti-ovarian tissue antibody levels in the serum of treated rats and in the normal and LPS-induced POF control groups. The results indicated that the ODs and calibrated concentrations at the cut-off endpoint were similar across all groups. This similarity confirms that the cut-off endpoints were appropriately selected for the raw ELISA data.

### Statistical analysis

The data are presented as the means ± standard errors of the means (SEMs). One-way ANOVA was used to analyse each parameter. Post hoc analysis was conducted via Tukey’s HSD test to compare the healthy control group with the positive control group and the positive control group with the treated group. All the statistical analyses were performed via SAS v20 (SAS, USA).

## RESULTS

Compared with normal rats, the LPS group exhibited a significantly greater immune response. This was evidenced by a much greater antibody titre (dilution at the cut-off endpoint) at both the second week (53.3 ± 10.7 vs 6.50 ± 1.50) and fourth week (85.3 ± 21.30 vs 13.05 ± 4.18), with a *P*-value of < 0.001. Similarly, the antibody index increased dramatically in the LPS group at two weeks (94.17 ± 5.59% vs 6.50 ± 0.11%) and four weeks (80.89 ± 4.35% vs 15.30 ± 5.00%), with *P*-values of < 0.001. Treatment with MP significantly suppressed the immune response provoked by LPS. The antibody titre in the MP-treated group was markedly lower than that in the untreated LPS group at two weeks (4.50 ± 1.26) and four weeks (6.50 ± 1.50) (*P* < 0.001). Concurrently, the antibody index was also significantly lower in the MP group than in the LPS group at two weeks (28.21 ± 8.05%) and four weeks (17.76 ± 4.92%) (*P* < 0.001).

The concentration of anti-ovarian antibodies in undiluted serum was measured in the LPS group (59.3 ± 15.6 ng/100 μl at week 2; 48.5 ± 46.5 ng/100 μl at week 4). Both the MP and secondary Ab treatments significantly reduced this concentration: compared with the LPS treatment, the MP treatment resulted in significantly lower concentrations at week 2 (42.01 ± 5.90 ng/100 μl, *P* < 0.05) and week 4 (33.40 ± 4.84 ng/100 μl, *P* < 0.05). Secondary antibody treatment resulted in an even greater reduction, with significantly lower concentrations at week 2 (31.22 ± 4.95 ng/100 μl, *P* < 0.05) and week 4 (18.6 ± 6.90 ng/100 μl, *P* < 0.01). Treatment with secondary antibodies also effectively reduced immune parameters. The antibody titre was significantly lower than that in the LPS group at both weeks (10.00 ± 2.00) and four weeks (14.00 ± 2.00) posttreatment (*P* < 0.001). Furthermore, the antibody index was significantly lower in the secondary Ab-treated group than in the LPS group at two weeks (45.7 ± 5.97%) and four weeks (50.30 ± 6.29%) (*P* < 0.05). The results indicated a strong autoimmune response in the LPS-induced rat model (LPS group), which was effectively mitigated by treatment with MP and secondary Abs. In contrast, the DEX-treated group showed a moderate reduction in AOA levels, although this difference was not statistically significant compared with the LPS group. The frankincense-treated group presented no significant reduction in AOA levels, indicating limited efficacy in suppressing the autoimmune humoral response.

Based on these data, induction of a POF rat model with LPS significantly disrupted hormone levels compared with the normal negative control group (Table 2). Rats treated with LPS (positive control) presented a severe hormonal imbalance characteristic of ovarian failure. Their serum 17β-oestradiol levels were dramatically lower (66.70 ± 4.82 pg/ml) than those of normal rats (136.15 ± 2.83 pg/ml), a difference that was highly significant (*P* < 0.001). Conversely, their gonadotropin levels were elevated, indicating a lack of negative feedback from the ovaries. Follicle-stimulating hormone (FSH) was significantly greater (9.43 ± 0.67 ng/ml vs 4.22 ± 0.82 ng/ml, *P* < 0.001), and luteinising hormone (LH) was also greater (11.881 ± 2.467 ng/ml vs. 6.124 ± 1.025 ng/ml, *P* < 0.05). Compared with the LPS-only group, all treatment groups showed a significant restorative effect on 17β-oestradiol levels, with levels restored to or even above normal levels. The LPS + methylprednisolone (MP) group presented the highest concentration (169.30 ± 3.90 pg/ml, *P* < 0.001), followed by the LPS + frankincense (138.69 ± 1.54 pg/ml, *P* < 0.001), LPS + dexamethasone (DEX) (134.51 ± 5.04 pg/ml, *P* < 0.001), and LPS + secondary antibody (121.52 ± 4.02 pg/ml, *P* < 0.001) groups. The treatments had varying effects on the elevated gonadotropins. For FSH, most treatments successfully lowered FSH levels back to the normal range. The levels in the LPS + frankincense (4.028 ± 0.410 ng/ml, *P* < 0.05) and LPS + secondary antibody (4.41 ± 1.98 ng/ml, *P* < 0.05) groups were not significantly different from those in the normal control group. The LPS + DEX group (6.71 ± 2.15 ng/ml) showed improvement, but the difference from the positive control was not statistically significant. Notably, the LPS + MP treatment appeared to increase FSH to a very high level (16.457 ± 0.33 ng/ml, *P < *0.001). All the treatments significantly reduced the elevated LH levels observed in the positive control. The LPS + DEX (5.461 ± 1.001 ng/ml, *P* < 0.05), LPS + secondary antibody (4.658 ± 0.964 ng/ml, *P* < 0.05), and LPS + MP (5.061 ± 1.124 ng/ml, *P* < 0.05) groups presented LH levels that were lower than those of the normal control. Compared with the positive control, the LPS + frankincense group (8.664 ± 2.754 ng/ml) also showed reduced LH levels, although this difference was not statistically significant. In summary, the LPS-induced POF model successfully created a hypogonadotropic state, which was effectively ameliorated by various treatments, particularly by restoring 17β-oestradiol and reducing LH levels. The FSH response to methylprednisolone was a notable exception and warrants further investigation.

**Table 2:** Concentrations of 17β-oestradiol 17β, FSH, and LH in the serum of a primary ovarian insufficiency (POI) rat model induced by LPS

Rat grouping	Oestradiol 17β (pg/ml)	FSH (ng/ml)	LH (ng/ml)
Normal rats (negative control)	136.15 ± 2.83	4.22 ± 0.82	6.124 ± 1.025
LPS-rats (positive control)	66.70 ± 4.82***	9.43 ± 0.67***	11.881 ± 2.467*
LPS + DEX	134.51 ± 5.04^c^	6.71 ± 2.15	5.461 ± 1.001^a^
LPS + secondary antibody	121.52 ± 4.02^c^	4.41 ± 1.98^a^	4.658 ± 0.964^a^
LPS + MP	169.30 ± 3.90^c^	16.457 ± 0.33^c^	5.061 ± 1.124^a^
LPS + frankincense	138.69 ± 1.54^c^	4.028 ± 0.410^a^	8.664 ± 2.754
			
*P*-values	<0.043 1	<0.013 2	<0.026 5

^a,c^Different lowercase letters indicate significant differences compared with the positive control group: *P* < 0.05, *P* < 0.01, and *P* < 0.001, respectively. The data are presented as the means ± standard errors (SEs). Asterisks denote significant differences compared with the negative control group: *P < 0.05, **P < 0.01, ***P < 0.001

DEX = dexamethasone; LPS = lipopolysaccharide; MP = methylprednisolone

### Ovarian tissue examination

**Figure 1:**
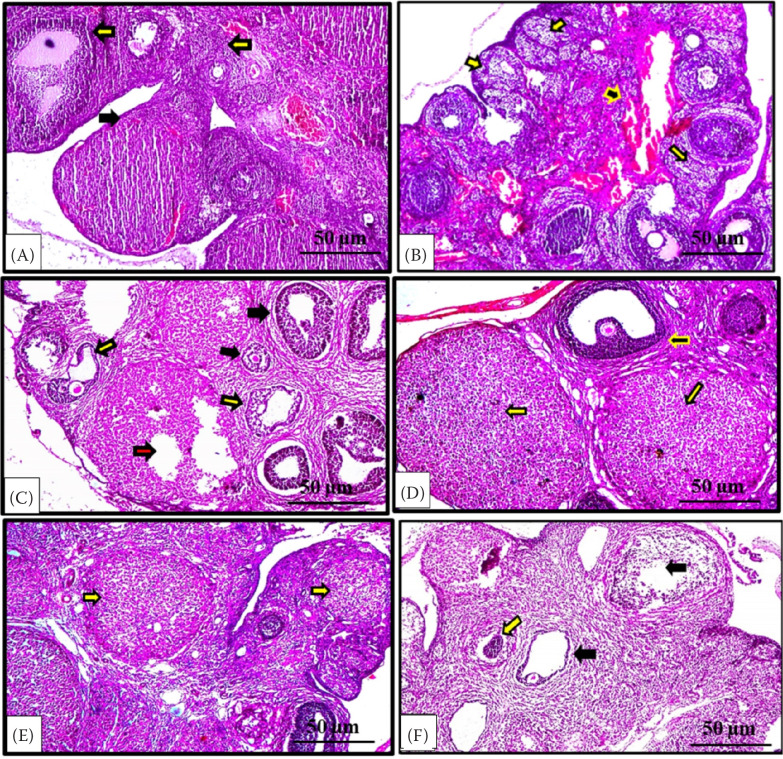
Histopathological examination of rat ovaries across experimental groups (A) Ovary from a rat in the negative control group, showing normal histology. The tissue exhibited well-developed corpora lutea (black arrows) and mature follicles at various developmental stages (yellow arrows). Moderate congestion is also present in the ovarian blood vessels. H&E stain; ×40 magnification. (B) The ovaries of the LPS-treated rats used as positive controls show multifocal areas of a group of atretic follicles in the ovarian tissue (yellow arrows) associated with severe degeneration in some other growing follicles (black arrows) via H&E staining; ×40 magnification. (C) The ovaries of LPS + DEX-treated rats showed few normal follicles at various stages (black arrows). Other spaces showed severe degeneration and folding in some other follicles (yellow arrows). Additionally, severe degeneration and necrosis of the lutein cells of the adjacent corpus luteum were observed (red arrow). H&E stain; × 40 magnification. (D) Ovary from a rat treated with LPS and a secondary antibody. The tissue appears completely healthy and active, with well-developed corpora lutea (yellow arrows) and mature Graafian follicles adjacent to them (black arrow). H&E stain; ×40 magnification. (E) Ovaries of the LPS + MP-treated rats showed moderate degeneration and necrosis in the lutein cells of multiple corpora lutea in the ovarian tissue (yellow arrows). H&E stain; ×40 magnification. (F) The ovaries of the LPS + frankincense-treated rats exhibited severe degeneration in some mature follicles (black arrows). Additionally, atrophy in some other follicles was observed (yellow arrow). H&E stain; ×40 magnification

The ovarian tissues of the normal rats (negative controls) showed normal histology and physiology, with many well-developed corpora lutea adjacent to one another. Among the corpora lutea, normal ovarian follicles at various stages were observed. Additionally, mild ovarian blood vessel congestion was observed (Figure 1A). The rats injected with lipopolysaccharide (LPS) and designated as positive controls had ovarian tissues with multifocal areas containing clusters of atretic follicles. In certain areas, there is evidence of severe degeneration and nuclear pyknosis in the inner cell layer of some other mature follicles, which is associated with ovarian blood vessel congestion and haemorrhages. Furthermore, ovarian tissue revealed evidence of the rupture of a fully mature follicle (Figure 1B). When POI conditions were treated with DEX as a therapeutic model, few normal ovarian follicles at various stages were found. Severe deterioration and folding in several follicles have been observed in various areas of the ovarian tissue. The corpora lutea’s lutein cells also exhibit severe necrosis and degeneration. Furthermore, severe desquamation, necrosis, and degeneration were noticeable in the oviductal villi of the nearby oviduct (Figure 1C). The POI treated with secondary antibodies as a treatment model fully recovered the physiological activity of the examined tissues. This included fully healthy and active ovarian tissues with multiple well-developed corpora lutea and mature Graafian follicles next to the corpora lutea (Figure 1D). When the POI model was treated with MP, the ovarian tissue, which exhibited considerable degeneration and necrosis of lutein cells in several corpora lutea, did not recover. In the core regions of the corpora lutea, nuclear pyknosis was also observed in the lutein cells, leaving a whitish hollow zone surrounding them (Figure 1E). The ovaries of the LPS + frankincense-treated rats exhibited severe degeneration in some mature follicles. Additionally, atrophy in some other follicles was observed (Figure 1F).

## DISCUSSION

The investigation presented herein examines the complex interplay among systemic inflammation, autoimmune reactivity, and ovarian function, offering a critical evaluation of therapeutic strategies for autoimmune-mediated POF. Using an LPS-induced rat model, this study successfully recapitulates a core feature of animal and human autoimmune ovarian insufficiency: the breakdown of immunological tolerance and the subsequent generation of anti-ovarian antibodies (AOAs). These findings demonstrate that LPS administration triggers a robust autoimmune response, as evidenced by significantly elevated AOA titres, a characteristic hormonal profile of hypergonadotropic hypogonadism (elevated FSH/LH with low oestradiol), and profound histopathological damage to the ovarian architecture. These results are consistent with a growing body of literature that implicates inflammatory insults as potential triggers for autoimmune endocrine pathologies, including ovarian failure (Ishizuka 2021; Lv et al. 2021). The model aligns with the hypothesis that infections or systemic inflammatory events can expose sequestered ovarian antigens or molecular mimics, thereby initiating an immune attack on follicular structures, particularly granulosa and theca cells, which are rich sources of potential autoantigens.

The central finding of this study is the superior efficacy of secondary antibody therapy over other interventions. This treatment modality achieved full remission: it normalised circulating AOA levels; restored a balanced hormonal milieu (E2, FSH, and LH); and, most importantly, facilitated complete histological recovery of the ovarian tissue. The presence of well-developed corpora lutea and mature Graafian follicles in this group indicates a return of ovulatory function and endocrine activity. This remarkable outcome suggests an exquisitely targeted mechanism of action. The secondary antibodies produced by immunisation against the primary AOA are postulated to function as anti-idiotypic antibodies. According to network theory, anti-idiotypic antibodies can bind to the variable region (idiotype) of a pathogenic autoantibody, effectively neutralising it and preventing its binding to ovarian antigens (Großerichter-Wagener et al. 2020). This approach offers a paradigm shift from nonspecific immunosuppression to targeted, biological neutralisation of the pathological agent itself. It halts autoimmune attack at the humoral level without broadly dampening the entire immune system, thereby minimising collateral damage and potential side effects. This strategy is gaining significant interest in various autoimmune conditions, from rheumatic diseases to myasthenia gravis (Gerischer et al. 2025), and our findings strongly support its application in reproductive immunology (Cuadrado-Torroglosa et al. 2024). Future work should focus on identifying the specific ovarian antigens targeted by AOAs in this model (e.g., zona pellucida proteins, steroidogenic enzymes, or receptors) to develop even more specific monoclonal anti-idiotypic therapies.

In stark contrast, results from corticosteroid interventions reveal a complex, paradoxical relationship between immunosuppression and ovarian recovery. MP demonstrated potent immunosuppressive capabilities, effectively suppressing the humoral immune response to the extent that AOA concentrations and titres were reduced to levels comparable to, or even lower than, those in the healthy control group. This aligns perfectly with the well-documented pharmacology of high-dose glucocorticoids, which induce lymphocyte apoptosis, inhibit B-cell activation and antibody production, and suppress proinflammatory cytokine release (Babaoglu and Karadag 2024). However, this powerful suppression of the antibody response profoundly differed from the functional and morphological outcomes. The MP group exhibited a failure to correct, and indeed a paradoxical exacerbation of, FSH levels, alongside persistent and severe histopathological lesions featuring degeneration and necrosis within the corpora lutea. This dissociation suggests several possibilities. First, the dose or regimen of MP used, while effective for antibody suppression, may have been directly toxic to ovarian follicles and granulosa cells, which express glucocorticoid receptors and are sensitive to steroid-induced apoptosis (Huang et al. 2023). Second, it is plausible that the autoimmune damage in this model is not solely antibody-mediated but involves a significant cell-mediated component (e.g., cytotoxic T cells) that is less responsive to this particular corticosteroid regimen. Antibodies may be biomarkers of disease rather than the sole effector mechanism. Third, intense immunosuppression may disrupt delicate cytokine networks necessary for normal folliculogenesis and ovulation. This finding is clinically crucial, as it echoes the ongoing debate and mixed outcomes observed in studies using corticosteroids to treat suspected immune infertility in women (Kwak-Kim et al. 2022). This finding cautions against the assumption that the suppression of a humoral marker is equivalent to curing the disease and highlights the potential for iatrogenic harm.

DEX treatment resulted in an intermediate phenotype. It was successful in restoring oestradiol production and normalising LH levels, indicating a positive effect on the steroidogenic function of theca and granulosa cells, perhaps through a general reduction in ovarian inflammatory stress. However, its impact on specific autoimmune parameters (AOA levels) was less robust than that of MP or secondary antibodies, and the degree of histological improvement was incomplete, with evidence of ongoing tissue degeneration. This finding suggests that DEX, at the administered dose, provided a partial anti-inflammatory benefit but was insufficient to fully eliminate the autoimmune response or reverse established damage. The differential effects of MP and DEX, despite both being corticosteroids, could be attributed to their varying pharmacokinetic and pharmacodynamic properties, including receptor affinity, half-life, and mineralocorticoid activity, which can influence their tissue penetration and overall immunological impact (Liu et al. 2024). The dissociation between the MP-induced suppression of AOA and the lack of histological or functional recovery suggests that the autoimmune pathology in this model may not be solely antibody-mediated. A significant cell-mediated immune component, such as cytotoxic T-cell infiltration or macrophage activation, could contribute to ovarian damage. Corticosteroids such as MP may fail to adequately target these cellular effectors, leading to persistent tissue injury despite reduced antibody titres. Future studies should investigate the role of T-cell subsets and cytokine profiles in autoimmune POF to better understand the full immunopathological spectrum.

The results from the frankincense (Boswellia) treatment group add a fascinating layer to the discussion, situating a traditional natural remedy within a modern immunological context. Frankincense extract, which is rich in boswellic acids, is a recognised natural anti-inflammatory agent that works primarily by inhibiting 5-lipoxygenase (5-LOX) and consequently the leukotriene pathway, as well as by modulating other proinflammatory mediators, such as TNF-α and IL-1β (Efferth and Oesch 2020; Meyiah et al. 2023). Its efficacy in normalising oestradiol and FSH levels is significant, suggesting that its broad anti-inflammatory action successfully mitigates inflammatory cascades that contribute to ovarian dysfunction in this model. Chronic inflammation is known to impair granulosa cell function and disrupt the hypothalamic–pituitary–ovarian axis (Deng et al. 2024). By reducing this inflammatory burden, frankincense may have created a more favourable microenvironment for residual follicles to function. However, its limitations were equally apparent: it had a minimal effect on the specific AOA titre and failed to produce significant histological recovery. These findings indicate that while the general inflammation initiated by LPS was ameliorated, the specific adaptive autoimmune response (AOA production) remained largely unchecked. This makes a strong case for the distinction between general anti-inflammatory therapy and targeted immunotherapy in autoimmune conditions. Frankincense may be better suited as an adjunctive therapy to manage background inflammation, or it could be explored for conditions of ovarian dysfunction driven primarily by inflammation rather than explicit autoimmunity. Notably, the use of crude frankincense powder, rather than purified boswellic acids or other active compounds, may limit the interpretability of its therapeutic potential. Future studies should employ standardised extracts to better evaluate their efficacy and mechanisms.

In addition to the direct comparison of therapies, this study reinforces the multifaceted pathophysiology of autoimmune POF. The histopathological observations provide a visual narrative of the disease process, from the multifocal atretic follicles and vascular congestion in the LPS group, indicating ischemia and immune attack, to the varying degrees of recovery and failure observed with each treatment. The complete restoration of architecture with secondary antibodies is the ideal outcome. Persistent necrosis with MP is a warning. The partial follicular atrophy with frankincense suggested that the damage was halted but not reversed. These histological endpoints are arguably as important as the serological endpoints, as they directly reflect the organ’s functional reserve.

From a clinical perspective, these findings have profound implications. They argue for a more nuanced diagnostic approach in women with idiopathic POF or poor ovarian response, one that includes screening for AOAs and other cytokine antibodies to identify an autoimmune subtype (Abo-Aziza et al. 2022; Federici et al. 2024). For those who test positive, the data suggest that nonspecific glucocorticoid therapy, while potentially beneficial for some, carries the risk of being ineffective or even harmful for others. The promise of targeted therapies such as anti-idiotypic antibodies offers a glimpse into the future of personalised reproductive medicine. While the translation from a rat model to human and animal therapy is complex and requires extensive further research, the principle of neutralising pathogenic antibodies is already a reality in other fields (e.g., the use of intravenous immunoglobulin or specific biologics).

In conclusion, this comprehensive study significantly advances our understanding of therapeutic strategies for premature autoimmune ovarian failure. This finding robustly confirms the pathogenicity of anti-ovarian antibodies in an inflammatory model and provides a clear hierarchy of treatment efficacy. It champions the targeted biological therapy paradigm, exemplified by secondary antibodies, which achieve complete immunological, hormonal, and histological restoration. Moreover, the use of broad-spectrum corticosteroids warrants caution, as potent immunosuppression does not guarantee functional recovery and may cause unintended harm. Finally, it positions natural anti-inflammatories such as frankincense as potential modifiers of the inflammatory landscape but not as a standalone solution for established autoimmunity. Future research must focus on identifying key ovarian autoantigens, developing monoclonal anti-idiotic antibodies, and designing clinical trials to translate these promising preclinical findings into effective and safe therapies for women and animals suffering from this devastating condition.
